# Advanced Imaging Techniques in Diagnosis of Posterior Reversible Encephalopathy Syndrome (PRES)

**DOI:** 10.3389/fneur.2020.00165

**Published:** 2020-03-11

**Authors:** Nasim Sheikh-Bahaei, Jay Acharya, Anandh Rajamohan, Paul E. Kim

**Affiliations:** Division of Neuroradiology, Department of Radiology, Keck School of Medicine, University of Southern California, Los Angeles, CA, United States

**Keywords:** MR spectroscopy (MRS), CT perfusion (CTP), positron emission tomography (PET), posterior reversible encephalopathy syndrome (PRES), MR perfusion, susceptibility weight imaging

## Abstract

Diagnosis of Posterior Reversible Encephalopathy Syndrome (PRES) in some circumstances can be challenging and structural imaging may not be sufficient to distinguish it from other differential diagnostic considerations. Advanced imaging techniques, such as MR spectroscopy or positron emission tomography (PET) can provide additional information to determine the diagnosis. Other techniques, such as susceptibility weighted imaging (SWI) improves detection of hemorrhage which has prognostic role. CT or MR Perfusion as well as Single-Photon Emission Computed Tomography (SPECT) are more useful to understand the underlying vasculopathic changes in PRES and may answer some of the unresolved controversies in pathophysiology of this complex disease. In this review we summarized the findings of previous studies using these advanced methods and their utilities in diagnosis or prognosis of PRES.

## Introduction

Despite all the new developments in the field of neuroimaging, diagnosis of PRES in atypical cases remains challenging. Utilizing advanced imaging techniques can help clinicians to exclude the mimics and provide a more accurate diagnosis at the earlier stage. Some of these methods can also provide insight into the complex pathophysiology of the disease. In this article we discuss the role and findings of these advanced imaging techniques in diagnosis of PRES.

## MR Spectroscopy

MR spectroscopy (MRS) provides valuable information about the brain chemicals and metabolites, neuronal and glial cells activity, cell membrane integrity and composition of the cells in the region of interest. This data can be used to differentiate PRES from other diagnoses (1), predict outcome (2), or potentially enhance our understanding about the pathophysiology of the disease (3). There is lack of large comprehensive studies on MRS changes in PRES and the current data are mainly from case reports.

**Figure 1:**
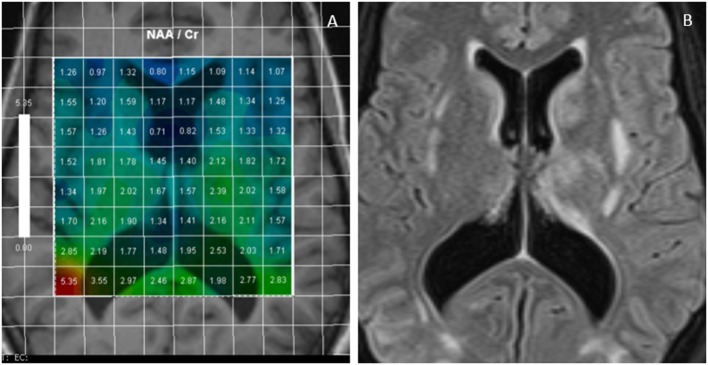
Multivoxel MR Spectroscopy in a PRES case shows reduced NAA/Cr in many regions such as periventricular white matter and around basal ganglia **(A)**; FLAIR images of the same case demonstrates foci of white matter hyperintensities **(B)**.

The main findings in most of the cases are reduction in the ratio of N-Acetylaspartate (NAA)/Creatine (Cr) (Figure 1) and NAA/Choline (Chol) (1–6). It has been claimed that this reduction is more due to increased Chol and Cr levels rather than a mild reduction in NAA. These alterations in the level of metabolites were seen beyond the boundary of T2/FLAIR signal changes and both in white and gray matters indicating the diffuse process in PRES (3). More interestingly, in some studies the level of NAA remained low and Chol high in the subacute phase of the disease (from 2 weeks to 2 months) despite normalization of the structural MRI and resolution of the clinical symptoms (1, 2, 4).

NAA is an amino acid that is mainly synthesized in the mitochondria of the neurons, axons, and dendrites. It is considered a marker of neuroaxonal viability, function, and density. Its reduction in PRES is more likely secondary to disruption of the synapses and neuroaxonal function rather than permanent loss of neurons or cell death as the changes are usually reversible with no evidence of atrophy at the later stage. Presence of vasogenic edema could also contribute to the mild reduction of NAA through dilution effect (3) with reduction of the density of neurons in each MRS voxel.

Chol is a marker of cell membrane turnover, inflammation, glial cell activation and demyelination. In PRES, increased in Chol peak is considered to be related to glial cell activation and also membrane synthesis in the subacute phase of the disease (1, 3).

There are contradictory results about the level of lactate (Lac) in PRES between different studies, as some detected increased peak of lac (2, 4) while others did not (1, 3). Know et al. presented four pediatrics cases with PRES and showed no changes in the level of NAA, Chol and Cr, but increased in Lac. Nearly all of them had complete remission in the follow up MRS exams (7). In adults and based on limited data, the peak of Lac is considered as a marker of tissue infarction rather than changes directly related to PRES (2).

Initial changes in the level of NAA, Chol, and Cr or even persistent changes in the subacute phase of PRES does not predict poor outcome but can be used to differentiate PRES from other mimicking pathologies, such as infarct, demyelination, encephalitis or tumor (8). Presence of Lac is in favor of infarct or other pathologies and could indicate permanent tissue damage. Increased ratio Chol/Cr is more favorable for tumor compared to PRES in which both Chol and Cr levels increase. Metabolic changes in demyelinating processes are usually restricted to white matter unlike PRES which are present in both gray and white matter (3).

## Susceptibility Weighted Imaging (SWI)

Hemorrhage is identified in 15–17% of PRES cases in large cohorts using Gradient echo (GRE), FLAIR or CT (9). However, recent studies have reported higher prevalence of hemorrhage (26–64%) in PRES using SWI (10, 11). There are three main types of hemorrhage in PRES: cerebral microbleeds (CMB, <5 mm), intraparenchymal hematoma (IPH, >5 mm) and subarachnoid hemorrhage (SAH) with or without intraventricular extension. CMB has become the most common type of hemorrhage identified in recent PRES studies due to superiority of SWI in detecting CMB. This may also explain the higher prevalence of total hemorrhage in the recent data (9, 10). CMB can hardly be seen in other sequences, such as FLAIR, T1, and T2. Moreover, there are several reports confirming the advantage of SWI compared to GRE or T2^*^ sequences in identifying CMB or small hemorrhages (12, 13).

Many studies have claimed that evidence of hemorrhage in imaging is associated with poor prognosis or fatal outcome (9, 11), while others did not show the same association (10). Hemorrhage is also associated with more severe T2/FLAIR signal changes and presence of cytotoxic edema and restriction in diffusion-weighted images (DWI) (14). Although the direct effect of CMB on prognosis of PRES is not completely understood, follow up studies (between 4 and 320 days) have shown that CMBs secondary to PRES remained unchanged even after normalization of the FLAIR signal (10), which could have long term implications.

Moreover, there are reports of PRES development secondary to cerebral amyloid angiopathy inflammation (CAA-I) (15). Presence of multiple CMBs on SWI, particularly if they were present prior to the latest onset of PRES, can suggest CAA-I as the cause of attack.

Susceptibility weighted angiography (SWAN) has also been used in PRES showing transient reduction in the susceptibility of the venous system at the initial phase of PRES, associated with hyperperfusion which was normalized by day 40 (16).

Based on current data, performing SWI will provide additional valuable information, which can be used to predict the outcome of PRES. However, longitudinal data are required to investigate the effects of CMB secondary to PRES in long term prognosis and their potential predisposition to other neurological disorders, such as hemorrhagic stroke or Alzheimer's disease in the future.

## CT and MR Perfusion

**Figure 2:**
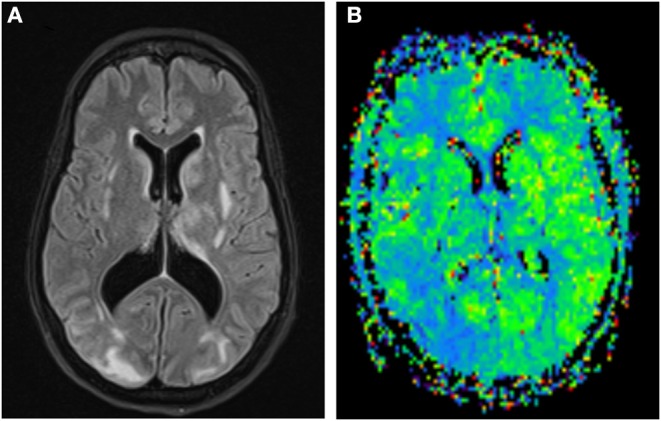
FLAIR images of a PRES case with white matter hyperintensities more prominent in the right occipital lobe **(A)**; MR Perfusion shows reduction in MTT in the right occipital lobe suggestive of hyperperfusion **(B)**.

The results of CT perfusion studies in PRES are contradictory. Some studies have found hyperperfusion with increased cerebral blood flow (CBF), cerebral blood volume (CBV) and reduction in time to peak (TTP) and mean transit time (MTT) (17–19) (Figure 2). Other studies showed opposite results with vasoconstriction, reduction in CBF, near normal CBV, and increased in TTP, MTT, and time to drain (TTD) (20–22).

The reports on MR perfusion follow the same pattern with conflicting results across different studies. Although many authors reported decreased CBF, CBV, and increased MTT (23–25), there are few reports of increased perfusion in PRES cases (26–28).

There are two main theories regarding the development of vasculopathy and vasogenic edema in PRES:

1. Severe hypertension exceeds the limit of autoregulation in vessels, leading to hyperperfusion, vascular dilatation, endothelial damage, leakage, and vasogenic edema.
2. The earlier original theory which was based on vasospasm and hypoperfusion as a compensatory mechanism against hypertension resulting in brain ischemia and consequently vasogenic edema (29).

Collective data in recent years however, might be more suggestive of a combination pathway with vasoconstriction and vasodilatation changes both present at the same time or sequential in the course of the disease. When the systemic blood pressure rises, the neurovasculature system attempts to maintain a constant flow by autoregulation, and arterioles constrict to create compensatory resistance and avoid hyperperfusion (30, 31). It has been shown in animal models that when the blood pressure exceeds the upper limit of autoregulation, the constricted arterioles forced to dilate and there is blood brain barrier disruption and extravasation of the fluid and red blood cells into the parenchyma (32). In addition, there is evidence of endothelial injury in the small vessels leading to endothelial thickening, occlusion of the small vessels, microbleeds, and hypoperfusion.

Although the abovementioned mechanisms can explain the underlying pathophysiology of vasculopathy in hypertension induced PRES, they cannot be generalized to all PRES cases as many of them present with normal or near normal blood pressure. A growing body of evidence suggests in clinical conditions presenting with PRES, there are similar systemic processes contributing to vasculopathy including activation of immune system, increased in cytokines and interleukins, endothelial damage with increased permeability, increased leukocyte adherence, and consequently microcirculatory dysfunction, focal vasoconstriction and vasodilatation and beading of the vessels and consequently tissue damage (21, 29).

The conflicting results in perfusion studies more likely reflect the complex pathophysiology of PRES and dynamic vascular changes during the course of the disease. The initial cause of PRES might also play a role. The time of imaging in relation to the onset of symptoms and also the start of antihypertensive therapy and intensity of treatment could significantly affect the result of perfusion studies. In a report by Casey et al., they found transient hypoperfusion in a PRES case treated aggressively with antihypertensive drugs. The hypo-perfused regions returned to normal after reducing the intensity of treatment and keeping the blood pressure above a certain level (30).

Although perfusion studies may not help the diagnosis or prognosis of PRES in day to day clinical practice, they can be used for better understanding of the pathophysiology of this complex and heterogeneous condition. Further larger studies with standard techniques on more homogenous cohort are required in acute, subacute and later phases of PRES to advance our knowledge about the patterns of vasculopathy and changes of perfusion in PRES.

## Single-Photon Emission Computed Tomography (SPECT) and Positron Emission Tomography (PET)

The most common type of SPECT used in PRES is Technetium-99 m-hexamethylpropyleneamineoxime (99 mTc-HMPAO). The results of SPECT in PRES are very similar to CT and MR perfusions with conflicting reports of hypo or hyperperfusion. Some studies found hypoperfusion in watershed areas and in regions of vasogenic edema on MRI (33, 34), while other reported hyperperfusion in T2 FLAIR hyperintense regions (26–28). There are also reports of using single-photon emission CT with N-isopropyl-(123)I-p-iodoamphetamine (IMP-SPECT) during recovery stages of PRES, showing normal uptake in most cases when MRI became normal (35, 36). Although hypoperfusion in IMP-SPECT at early follow up (11 days) (34) or focal hyperperfusion in a case with persistent MRI changes after 30 days (36) were also reported. (133)Xe-SPECT has also been used to assess perfusion in PRES and showed low uptake in areas of vasogenic edema (36).

Data is limited on the utility of PET in PRES. In most of studies, the whole body PET was performed for diagnosis of the underlying cancer or to detect metastases.

The first report of using (18 F) fluorodeoxyglucose (FDG)-PET in PRES was on a young boy with systemic lupus erythematosus. They found hypometabolism in occipital-parietal region where T2 hyperintensity and hemorrhage were present (37). Rath et al. used FDG and (11 C) methionine (MET)-PET to differentiate atypical unilateral PRES from low-grade tumor, showed decreased uptake of FDG and minimal uptake of MET in the regions of MRI abnormality in PRES (38). Brain Gliomas on the other hand have high MET uptake due to increased metabolic rate and their uptake ratio is associated with the tumor viability (39). Although many studies have found hypometabolism in FDG, there is a report of increased FDG uptake in the regions of T2 FLAIR hyperintensities in a pediatric patient with Burkitt's lymphoma presented with PRES (40).

FDG-PET uptake usually has close association with the MRI changes, it becomes normal at the later stage of PRES when the FLAIR signal normalizes (41) but remains low with persistent MRI changes in more severe cases (42).

(11 C) Pittsburgh compound (PiB) and (18 F) FDG-PET were also used on a PRES case secondary to CAA-I. High amyloid uptake in PiB and focal hypometabolism in FDG was found in the regions of FLAIR/T2 hyperintensity (43).

Based on limited data available, utility of PET in PRES is mainly to distinguish it from tumor. Low FDG and MET uptake can differentiate the two that is particularly useful in cases with unilateral or marked asymmetric changes mimicking mass in the structural imaging.

## Discussion

When the clinical presentation of the PRES is unusual or there are atypical changes in the structural imaging, advanced imaging techniques can help in distinguishing PRES from mimics. Table 1 summarizes the imaging features of PRES in different advanced imaging techniques.

- MRS can help differentiating PRES from low grade tumor, demyelination or other encephalitis.
- SWI is the best sequence to detect CMB and can significantly improve the rate of hemorrhage detection. The knowledge about load and number of persistent CMB after the onset of PRES would provide valuable information, which may have potential long-term implication.
- There are contradictory results about perfusion changes in PRES. These studies can be used for better understanding the disease pathophysiology rather than diagnosis of PRES. The conflicting results are most likely secondary to dynamic vascular changes during the course of the disease, complex pathophysiology with variations across different underlying causes, timing of the perfusion studies, and the use of antihypertensive treatments. To address these issues there is a need for larger cohort studies using more standard methods, alleviating some of the co-founding factors.
- MET and FDG PET can also be used to differentiate PRES from low grade glioma.

**Table 1:** Summary of imaging features using different advanced imaging techniques in PRES.

**Imaging techniques**	**Findings**
MRS	- Reduction in NAA/Cr and NAA/Chol. - Lac may or may not be present. Presence of Lac might represent infarcted tissue. - Metabolite changes are not limited to only white matter and can be even detected in regions with normal T2/FLAIR signal.
SWI	- Improves the rate of hemorrhage detection. - Pre-existing microbleeds in CAA can be a risk factor for future PRES. - Microbleeds secondary to PRES are persistent even after normalization of the FALIR signal.
Perfusion	Both - Hyperperfusion (increased CBF and CBV with reduced TTP and MTT). - Hypoperfusion (reduced CBF and CBV with increased MTT) have been reported. - The cause of PRES and administration of antihypertensive medication can influence the result of perfusion study.
PET/SPECT	- Low FDG and Met uptake in most PRES cases. - Results of HMPAO-SPECT is similar to CT/MR perfusion (both hypo- and hyperperfusion state have been reported.)

*CAA, cerebral amyloid angiopathy; CBF, cerebral blood flow; CBV, cerebral blood volume; Chol, choline; Cr, creatine; HMPAO, hexamethylpropyleneamineoxime; Lac, lactate; Met, methionine; MRS, MR spectroscopy; MTT, mean transit time; NAA, N-Acetylaspartate; PET, Positron Emission Tomography; SPECT, Single-Photon Emission Computed Tomography; SWI, Susceptibility Weighed imaging; TTP, time to peak*.

## Author Contributions

All authors listed have made a substantial, direct and intellectual contribution to the work, and approved it for publication.

### Conflict of Interest

The authors declare that the research was conducted in the absence of any commercial or financial relationships that could be construed as a potential conflict of interest.
